# Contributions of DNA mechanics and trans-regulation to nucleosome positioning in *Schizosaccharomyces pombe* and its role in co-transcriptional splicing

**DOI:** 10.1186/s13072-026-00675-0

**Published:** 2026-04-21

**Authors:** Guoqing Liu, Jing Cang, Zhihao Du, Xiangjun Cui, Hongyu Zhao, Jia Liu

**Affiliations:** 1 School of Life Science and Technology, Inner Mongolia University of Science and Technology, Baotou, 014010 China; 2 Inner Mongolia Key Laboratory of Life Health and Bioinformatics, Inner Mongolia University of Science and Technology, Baotou, 014010 China; 3 School of Science, Inner Mongolia University of Science and Technology, Baotou, 014010 China

**Keywords:** DNA deformation energy, Rotational positioning, Gene expression, Splice site, 3D genome

## Abstract

**Background:**

Nucleosome positioning critically regulates chromatin functions, yet species-specific mechanisms remain incompletely understood. This study revisits nucleosome organization in *Schizosaccharomyces pombe (S. pombe*) using a DNA deformation energy model and a high-resolution nucleosome map.

**Results:**

We demonstrate that DNA bending energy—not shearing energy—accurately predicts rotational positioning (72.2–77.4% accuracy) and nucleosome-depleted regions (NDRs) near transcription start sites (TSSs) in *S. pombe*. Gene-end analyses reveal that NDRs and nucleosome phasing are, at least partly, encoded in DNA sequence. Strikingly, nucleosome enrichment at RNA splice sites is determined primarily by trans-acting factors (e.g., transcription factors Pcr1/Atf1), not by DNA sequence preference for nucleosome positioning, and correlates with splice site usage rates. Highly transcribed genes exhibit reduced nucleosome occupancy upstream of splice sites, while frequently used splice sites show elevated nucleosome occupancy. Furthermore, 3D chromatin architecture analysis indicates that highly transcribed intron-poor genes display enhanced medium-range chromatin looping (10–100 kb), potentially reflecting their preferential aggregation at sub-nuclear environments enriched in transcriptional machinery and splicing factors.

**Conclusions:**

Our work identifies DNA bending properties as an important contributor to *S. pombe* nucleosome organization and reveals the involvement of nucleosome positioning and chromatin architecture in co-transcriptional splicing.

**Supplementary Information:**

The online version contains supplementary material available at 10.1186/s13072-026-00675-0.

## Background

Nucleosome is the basic repeating component of chromatin in eukaryotes, and plays essential roles in chromatin functions, such as gene transcription, DNA replication, and recombination, consequently modulating cell behaviors [25]. For example, nucleosome depletion at or near the transcription start sites was revealed to favor transcription [57]. Nucleosomes were also depleted at replication origin sites and recombination hotspots [39, 52]. Nucleosomes also contribute to RNA Polymerase II pausing, thereby regulating transcription [10, 67]. Nucleosomes at some regions, such as transcription start sites (TSSs) of some developmentally crucial genes and target sites of developmentally important transcription factors, could be displaced during cell differentiation [64]. Inducible activation of promoters is associated with transient nucleosome depletion only at alleles undergoing transcription initiation [50]. Interesting species-specific nucleosome positioning patterns were reported [47], but underlying mechanisms remain under-investigated. The factors governing nucleosome positioning generally fall into two categories: intrinsic DNA-dependent factors and extrinsic factors. For example, the presence of characteristic dinucleotides separated 10 bp away from each other on nucleosome core DNA was confirmed to assist DNA wrapping around histones [57], (Struhl and Segal, [61]). Extrinsic factors include remodelers, transcription machinery, histone modification, histone chaperone, and histone variants [24, 29, 37, 40, 45, 46, 70, 73]. Although great progress has been made in understanding how nucleosome positioning is regulated [53, 57], (Struhl and Segal, [61]), [66], some unexpected phenomena have drawn considerable interest. For example, it was reported that *Schizosaccharomyces pombe* (*S. pombe*) differs remarkably in nucleosome positioning pattern from *Saccharomyces cerevisiae* (*S. cerevisiae*): *S. pombe* has abundant nucleosomes with much higher enrichment of A/T base and poly (dA-dT) tracts around dyad positions than *S. cerevisiae*,*S. pombe* has deeper nucleosome depleted regions (NDRs) near TSSs than *S. cerevisiae* [47],* S. pombe* NDRs are not enriched with poly (dA-dT) tracts, a stereotypical marker of *S. cerevisiae* NDRs [26]. What characteristics does nucleosome positioning in *S. pombe* have from the perspective of DNA energetics? We address this question in this study by utilizing our previously developed DNA deformation energy model [33, 35]. In our previous studies, DNA bending was shown to be an ideal predictor of the rotational positioning of nucleosomes in budding yeast and mouse [31, 33, 34, 35, 36], but it possible roles in fission yeast have not been systematically investigated. Furthermore, shearing energy-based models, which were more effective in estimating nucleosome occupancy in budding yeast, did not work in mouse [36]. In contrast, nucleosome occupancy in mouse ESCs could be predicted with a bending energy-based model [36]. These suggest that the species-specific nucleosome positioning mechanism might also be coupled to DNA deformation properties. We also revisit some other relevant issues, particularly the nucleosome positioning pattern around RNA splice sites in *S. pombe* and its possible determinants. Regarding experimentally determined nucleosome maps, it is worthy of noting that the base-pair resolution nucleosome map generated by a chemical cleavage approach, which can avoid sequence cleavage bias that MNase-based mapping has [11, 14, 15, 42] would facilitate our accurate analysis. Fenton reaction-based chemical approaches were successfully applied to yeast and mouse [8, 11, 67], and we have further explored the DNA physical property-dependence of nucleosome positioning based on the generated base-pair resolution nucleosome maps combined with DNA deformation energy models [31, 33, 34, 35, 67]. The performance of DNA physical properties in classifying nucleosome-enriched from -depleted regions was also demonstrated via a machine-learning approach [32]. Although several studies have reported the role of nucleosome positioning in RNA splicing [20, 21, 66], the effect of nucleosomes on alternative splicing remains poorly understood, and none of these studies are based on the highly accurate splice sites identified by direct RNA sequencing (dRNA-seq) on Oxford Nanopore Technologies (ONT) platforms. Nanopore dRNA-seq can produce reads covering up to full-length gene transcripts, allowing us to accurately resolve isoforms in full length. In this study, we utilize the highly accurate alternative splice sites identified by Nanopore dRNA-seq [44] to explore the role of nucleosomes in splicing.

## Materials and methods

### Gene annotation data

Gene annotation files (gff files) and genome sequence for *S. pombe* is obtained from PomBase database (https://www.pombase.org/data/genome_sequence_and_features/OLD/20131111/). Additionally, the annotation file for high-confidence transcripts was obtained from Lantermann et al. [26]. The high-confidence gene set comprises 3,693 genes, while PomBase-annotated set of all transcripts includes 6,835 genes, comprising both protein-coding genes and non-coding genes such as tRNAs, rRNAs, and ncRNAs. A comparative analysis revealed that all 3,693 high-confidence transcripts correspond to protein-coding genes. However, the genomic coordinates of genes in the two annotation sets are not entirely consistent. Specifically, 88 genes show exact matches in both start and end positions between the two files. Among the remaining genes, 3,058 of the 3,693 high-confidence transcripts are fully contained within genes annotated in PomBase, while 53 PomBase-annotated genes are fully contained within high-confidence transcripts.

### Experimental nucleosome maps of *S. pombe*

Chemical cleavage-based nucleosome map of *S. pombe* was taken from the previous study [47]. To be specific, both unique nucleosomes and redundant nucleosomes defined in the study were downloaded (supplemental Dataset S1 and Dataset S2 of [47]). Based on the nucleosome center positioning (NCP) scores in the redundant map, every nucleotide position of the genome was assigned a nucleosome occupancy score, which was the average value of NCP scores within a 121-bp window centered on that position. Finally, nucleosome occupancy values were zero-centered by subtracting genome-wide average.

MNase-determined nucleosome maps for two transcription factor (TF) mutants deficient in either *pcr1* or *atf1* were downloaded from GEO (GSE41773). To be specific, wig files of nucleosome occupancy were downloaded, and then linear interpolation was performed to obtain nucleosome occupancy scores at every genomic position.

MNase-determined nucleosome maps for two biological replicates for *snf21* switch-off mutant and a wild-type control of fission yeast were downloaded from GEO (GSE84912, [72]). The wig files of nucleosome occupancy were downloaded, and then data for two replicates were averaged. The chemical maps of nucleosome positioning for *S. cerevisiae* and mouse ESCs were obtained from the published studies [8, 67]. In the original studies [8, 47, 67], unique nucleosome maps of *S. cerevisiae*, *S. pombe*, and mouse ESCs were generated following the method developed by Brogaard et al. [8]. In brief, cleavage patterns around the nucleosome dyad were identified, and then a Poisson distribution model and a deconvolution method were used to compute the NCP score at every genomic position. Next, the noise level within the 147 bp region surrounding each genomic position was computed. Finally, a greedy algorithm was used to call nucleosomes sequentially based on the magnitude of NCP/noise signals. In the unique maps, nucleosomes are characterized by high NCP/noise signals and spacing above a defined distance threshold. For within- and between-species comparisons, we selected the top and bottom 10,000 nucleosomes from the unique maps based on their NCP/noise signals, and extracted their corresponding 500-bp sequences centered at nucleosome center positions from the reference genomes.

### Gene expression level and usage rates of splice sites

Transcription levels of *S. pombe* genes determined by dRNA-seq were derived from [44]. The genes were divided into four equal-sized groups according to quartiles of expression levels, and denoted as bottom 25%, bottom 25–50%, top 25–50%, and top 25%.

In the analyses considering different lengths of introns and exons, nucleosome occupancy profiles were generated using deepTools [55]. Signal matrices were computed with computeMatrix in reference-point or scale-regions mode. The latter normalizes intron regions to the same length of 100 bp to enable comparison across introns of different lengths. Profiles were then plotted using plotProfile.Splice site usage rate was defined by leveraging the isoforms detected by dRNA-seq [44]. The usage rate of a splice site is defined as the proportion of transcripts that utilize that site for splicing among all isoforms of a gene. For example, consider a gene with two isoforms expressed at fractions of 0.6 and 0.4. A 5' splice site may have a usage rate of 0.6 if it is exclusively used for splicing in the 0.6-fraction isoform, 0.4 if exclusive to the 0.4-fraction isoform, 1.0 if used in both isoforms, or 0 if not used in any isoform.

### Hi-C data processing

The Hi-C data of fission yeast were derived from GEO (GSE143338). The data includes two biological replicates of Hi-C data of fission yeast G2-arrested cells in control condition or in condensin shut-off condition. Condensin shut-off cells were obtained by depleting Cut14, which was a subunit of condensin known to shape 3D genome structure in *S. pombe* [22]. Chromatin interaction matrix files (.hic files) were downloaded from GEO (GSE143338). HiCExplorer (version 3.7) was used to process Hi-C data in this study [70]. Specifically, hicConvertFormat, a tool of HiCExplorer, was used to convert.hic files to.cool files with a bin size of 1000 bp. Hi-C contact matrices from two biological replicates were merged using hicSumMatrices. To enable a fair comparison between the wild-type and *cut14*-mutant strains, hicNormalize was applied to scale the merged matrices to the same total sum (based on the smallest library size), thereby removing differences in sequencing depth. Subsequently, hicCorrectMatrix was used with the Knight-Ruiz (KR) algorithm to correct for technical biases in GC content, mappability, and restriction enzyme efficiency. Based on the corrected matrices, hicAverageRegions was used to compute the averaged Hi-C contacts around 5' splice sites (5'SS) and 3' splice sites (3'SS), with a spanning range of 40 bins. The results were then visualized by hicPlotAverageRegions. To assess contacts between splice sites and between TSSs, hicAggregateContacts was used to aggregate 31-bin sub-matrices centered on the corresponding interaction foci in the Hi-C contact map, considering strand direction. Note that each cell of a heatmap generated by hicAggregateContacts represents aggregated contacts (observed/expected matrix) in a certain range (10–100 kb in this study), which differs from the averaged bin-pair contacts generated by hicAverageRegions.

### Calculation of DNA deformation energy and nucleosome occupancy

We used our previously developed DNA deformation energy model [33, 35] to investigate sequence-dependent properties of nucleosome positioning. The model calculates the elastic energy required to deform a given 129-bp DNA sequence into a predefined position-dependent structural template derived from high-resolution nucleosome crystal structures. In brief, a symmetric position-dependent template structure was created by averaging base-pair step parameters (roll, tilt, twist, shift, slide, and rise) from canonical nucleosome structures. Then the equilibrium structure for a query sequence was represented using dinucleotide-dependent equilibrium structural parameters. Finally, the deformation energy between the template and equilibrium structures was computed based on a harmonic model. Based on the deformation energy, nucleosome occupancy was estimated via Boltzmann law. The model successfully predicts free energy of nucleosome reconstitution in vitro, and rotational positioning of nucleosomes in *S. cerevisiae*. Rotational deformation energy, defined by three structural parameters (roll, tilt, twist), is associated with DNA bending properties [56] and proves powerful in predicting nucleosome rotational positioning [33, 35]. Our aim is to investigate the role of DNA bending property in nucleosome positioning of *S. pombe*. Therefore, unless stated otherwise, rotational deformation energy was used to predict nucleosome rotational positioning and occupancy throughout this study.

DNA shearing energy in nucleosome formation was calculated as described by Liu et al. [31]. In brief, shearing energy refers to the elastic deformation energy associated with the shear deformation of nucleosomal DNA along the superhelix axis. According to the study [31, 34, 56], DNA shearing arises primarily from two degrees of freedom: slide and shift. Intuitively, eliminating these two degrees of freedom (i.e., setting slide and shift to zero) would reduce the superhelical path of nucleosomal DNA with constant twist to a planar circle, devoid of axial shear deformation [5]. The computational details for the shearing energy were described in Liu et al. [31].

### Bi-class prediction model and feature importance analysis

We employed Random Forest, which is an extensively used ensemble learning algorithm [7], to discriminate strong nucleosomes from weak nucleosomes. The classifier was built as follows. Firstly, each nucleosomal sequence was represented as a feature vector of various DNA physico-chemical properties. The details of feature extraction were described in later. Then both positive (e.g. strong nucleosomes) and negative samples (e.g. weak nucleosomes) are partitioned into two equal-sized datasets, and hold-out validation was performed. Random Forest is successful in increasing the diversity of decision trees by random sampling of feature subset from the whole feature space at each splitting node. Based on the results of decision trees in the Random Forest, majority voting strategy is used to predict the class of a sample in the test set. We used R package “randomForest” to construct the Random Forest classifier. Based on our previous experience [33, 35], the number of decision trees in the Random Forest was set to 130, and the number of features sampled from the feature space at each splitting point was set to ${\mathrm{log}}_{{2}} m$, where *m* is total number of features in feature space. Area under the ROC curve (auROC) was used to evaluate the model performance. This procedure was applied to both within-species predictions (strong vs. weak nucleosomes) and between-species predictions (species A vs. species B). Feature importance was assessed by Gini index.

DNA physico-chemical properties include DNA shape parameters [76], DNA force constants [33, 35], DNA cyclizability [27], DNA flexibility for central step of tetranucleotide [51], chemical shifts for DNA backbone atoms (Table S1), three DNA deformation energies including shearing energy, bending energy and total deformation energy [31, 34], DNA rotational energy and translational energy [33, 35]. DNA shape parameters were calculated at base pair step resolution using R package DNAshapeR [13]. DNA chemical shift parameters are compiled from BMRB database (https://bmrb.io/).

Aforementioned sequence-based parameters were calculated for each base-pair or base-pair step. Then, mean, variance, and autocorrelations of the sequence-based parameters along the central 150 bp were calculated and used as final features in the prediction. Variance measures the variation of sequence-based parameters along the sequence, and autocorrelation captures long-range correlation. Both Moran and Geary autocorrelations of each parameter were calculated. The range of the lag parameter (lag) is set to 1–10.

## Results and discussion

### Sequence-dependent bending energy dictates rotational positioning

**Fig. 1:**
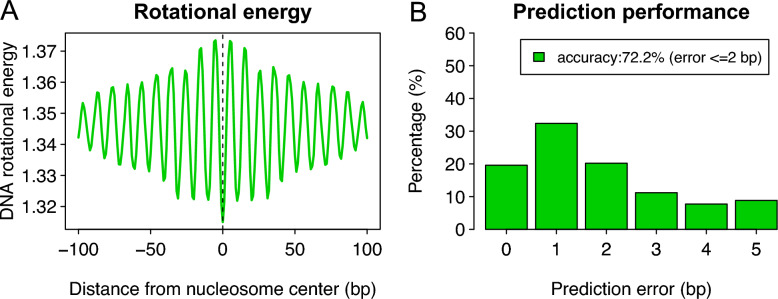
Prediction of rotational positioning of unique nucleosomes in *S. pombe* based on DNA rotational energy. The prediction error denotes the distance between the experimentally-determined nucleosome center position and the position with the minimum rotational energy in the interval [− 5, + 5] around the experimental nucleosome center. The rotational energy profile for a nucleosome typically shows a 10-bp periodic oscillation, and thus the interval [− 5, + 5] is large enough to cover all possible rotational positioning scenarios and have only one energy minimum. The y-axis shows the percentage of prediction error

In this study, we focused on the roles of DNA bending in nucleosome positioning of *S. pombe*. Since the deformation energy model [33, 35] depends on DNA physical properties, it can be directly applied to fission yeast without any species-specific training. To be more precise, DNA rotational energy defined in the model [33, 35] is used to quantify DNA bendability. When the model is applied to all unique nucleosomes of *S. pombe* [47], local rotational energy minima coincide very well with the nucleosome center positions (Fig. 1A). Because DNA helix always faces the histone octamer with its major groove at nucleosome center position, the match of energy minima with nucleosome center directly demonstrates that the model is successful in predicting rotational positioning. Rotational positioning refers to the specific orientation of DNA base pairs relative to the histone octamer surface in a nucleosome. It describes how the DNA helix is “rotated” around its own axis as it wraps around the histone core, determining which face of the DNA (major groove vs. minor groove) contacts the histone proteins.

The model accurately predicted the rotational setting of 72.2% unique nucleosomes in *S. pombe* with less than 2-bp uncertainty (Fig. 1B). When the prediction was confined to top 10,000 nucleosomes with highest NCP/noise signals, prediction accuracy increased to 77.4% (Fig S1).

### Nucleosome organization around gene ends

Nucleosome organization around gene ends is an extensively studied topic because of its special pattern and regulatory role in gene transcription. In order to explore the sequence-dependent property of nucleosome positioning in fission yeast, we first compared our bending energy-based nucleosome occupancy prediction and experimental map around gene ends. Bending energy is a good predictor of rotational positioning, and the latter constrains translational positioning. This rationalizes the bending energy-based model for nucleosome occupancy prediction. Before presenting results, let’s first explain the rationale. Rotational positioning establishes “allowed” frames wherein DNA can optimally contact histones, requiring specific roll/tilt angles at specific helical phases. This creates a preferred rotational frame every ~ 10 bp. Translational positioning selects among these rotationally-equivalent sites. Genomic regions with strong bending anisotropy preferentially bend toward a specific orientation to wrap around histones, exhibiting pronounced 10-bp periodicity in A/T- or G/C-rich dinucleotides that facilitate DNA curvature on the histone octamer surface. From the perspective of DNA mechanics, such DNA regions display strong 10-bp periodicity in bending energy profiles calculated using a sliding window (e.g. 129 bp) with a step size of 1 bp. This kind of DNA region is favorable for nucleosome formation with the major groove at the dyad position always facing toward histones, thereby constraining translational positioning. Conversely, DNA sequences with weak 10-bp periodicity in bending energy and high bending energy are unfavorable for nucleosome assembly.

**Fig. 2:**
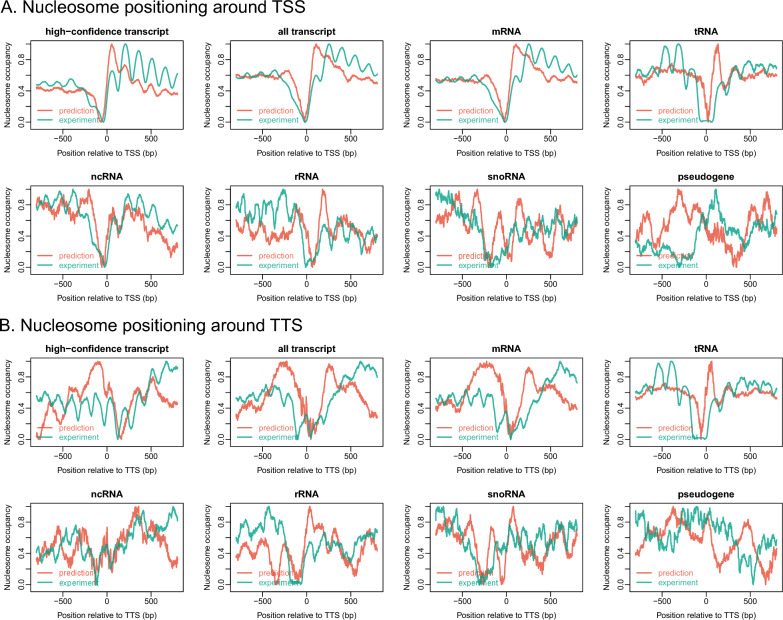
The comparison between chemical nucleosome map and deformation energy-based prediction around gene ends. (**A**), around TSS; (**B**) around TTS. Annotations for high-confidence transcripts were obtained from [26], and all other annotations for different types of genes were derived from PomBase database. For direct comparison, min–max normalization was applied to both experimental and predicted nucleosome occupancy profiles, rescaling values to the [0, 1] range. Experimental nucleosome occupancy was computed using the data from Moyle-Heyrman et al. [47]

For high-confidence transcripts in *S. pombe* annotated in a previous study [26], predicted nucleosome occupancy pattern shows a good agreement with the experiment (Fig. 2A). Both NDR near TSS and downstream nucleosome arrays are successfully reproduced by our model. When applied to all the annotated transcripts (including predicted transcripts and experimentally validated transcripts) in *S. pombe*, NDR near TSS was reproduced, but nucleosome phasing (regular nucleosome arrays) downstream TSS is not evident (Fig. 2A).

We further evaluated how nucleosome occupancy regulates transcription of specific types of genes (Fig. 2). Experimental nucleosome mapping showed that mRNAs, small nucleolar RNAs (snoRNAs), rRNAs and tRNAs have NDRs nearby their TSS, although some discrepancy exists in their position relative to TSS (Fig. 2A). These NDRs are successfully predicted by our model (Fig. 2A). Similar analyses for transcription termination sites (TTSs) show that nucleosome depletions near TTS of genes (such as genes encoding mRNAs, rRNAs and tRNAs) are also observed in both experimental map and prediction results (Fig. 2B). NDR is present at TSS of ncRNA, but absent at their TTS (Fig. 2). There is a broad NDR at the TSS of tRNA, and nucleosome arrays are arranged at both sides of the NDR (Fig. 2). A transcriptional directionality mechanism was proposed to explain the TSS-downstream nucleosome ordering [26], in which nucleosome arrays are laid out in the same direction as transcription, starting from the NDR adjacent to the TSS. It is possible that the TSS-upstream nucleosome array of tRNA genes reflects the presence of bidirectional promoters immediately upstream of those genes. To test this, we re-analyzed the tRNA genes by dividing them into two groups—those characterized by bidirectional promoters and those without. The results show that both groups exhibit upstream nucleosome arrays (Fig S2). This indicates that not all the nucleosome arrays in *S. pombe* can be explained by the transcriptional directionality mechanism. Interestingly, the predictions differ greatly from experimental occupancy for snoRNAs and pseudogenes (Fig. 2). As a negative control, pseudogenes exhibit no clear NDR at their ends. Most pseudogenes are degenerated non-functional copies of genes, and remain transcriptionally inactive [31, 34]. Therefore, there is no need to use organized nucleosome pattern to regulate their transcription (Fig. 2A).

Our results demonstrate that NDRs at gene ends, particularly at TSS, can be predicted by our DNA deformation energy model. Moreover, the genome-wide average nucleosome phasing around TSS in *S. pombe*—especially for high-confidence genes—can be recaptured by our sequence-based model (Fig. 2A). This indicates that NDR formation and nucleosome phasing around TSS depend, at least in part, on intrinsic DNA mechanical properties. To illustrate this, we provide examples where some of the experimental occupancy peaks in regular nucleosome arrays flanking specific gene TSS match our predictions (Fig S3). Genome-wide nucleosome positioning arises from the combined effects of intrinsic sequence preferences and extrinsic factors such as chromatin remodelers, histone variants and DNA methylation [9, 24, 29, 40, 46, 54]. Notably, sequence preferences may play a greater role in lower eukaryotes than in higher organisms, where nucleosome positioning determinants are more complex. Our results suggest that at least a baseline DNA-encoded nucleosome positioning signal is present in TSS-proximal regions. Note that our results do not contradict the well-established role of other important non-DNA factors, such as chromatin remodelers, in nucleosome phasing [17, 46, 49].Our previous attempt suggested that total deformation energy-based nucleosome occupancy prediction around TSS in *S. pombe* did not coincide well with experimental results [33, 35]. In addition, both translational energy-based model [33, 35] and shearing energy-based model [31, 34] also fail to predict the nucleosome pattern around TSS (Fig S4A). These indicate that DNA bending, rather than DNA total deformation or shear, dominates the nucleosome positioning around TSS and TTS in *S. pombe*. To show the superiority of our model, we also predicted nucleosome occupancy around TSS using two well-known models [23, 71]. There is a clear discrepancy between the predicted nucleosome pattern and the experimental map (Fig S4A-C).

Our results have another important implication: our model is totally DNA deformation property-dependent, regardless of A/T or G/C content, thereby suggesting that previously reported co-occurrence of A/T content and nucleosome occupancy peak in *S. pombe* [47] could be attributed to DNA bending ability. This also implies that DNA bending energy can be low (thereby favoring nucleosome formation) even if A/T is rich but properly phased in the sequence. The agreement between experimental nucleosome organization around TSS and prediction results is suggestive of the role of evolutionary shaped sequence-encoded nucleosome exclusion at their transcription initiation sites. Taken together, our results suggest that, in *S. pombe*, nucleosome depletion at gene ends and nucleosome phasing downstream TSS are regulated, at least partly, by DNA rotational energy property.

**Fig. 3:**
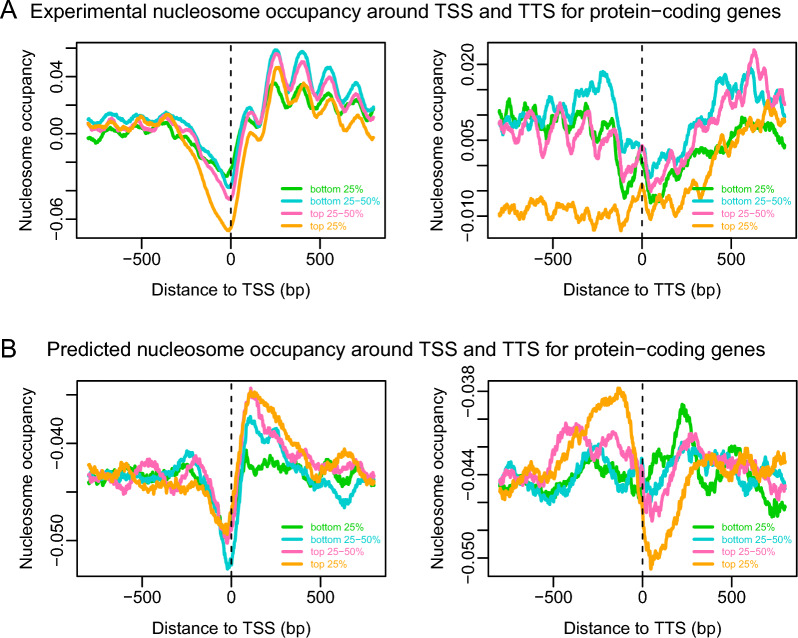
Nucleosome positioning pattern around TSS and TTS for protein-coding genes is associated with their transcription levels in *S. pombe*. (**A**), experimental nucleosome organization based on the data from Moyle-Heyrman et al. [47]. (**B**), predicted nucleosome organization

We also analyzed the relationship between the nucleosome patterns of protein-coding genes and transcription levels determined by dRNA-seq [44]. The four groups of genes divided by quartiles of transcription levels show characteristic nucleosome organization patterns at TSS (Fig. 3A). Specifically, consistent with previous results [47], highly transcribed genes have deeper NDR near TSS. For highly transcribed genes, NDR disappeared near TTS and the upstream wide range of TTS has a low level of nucleosome occupancy. It is possible that this pattern might be beneficial to RNA pol II elongation and increased transcription. We can also see that the TSSs of four gene groups are characterized with predicted nucleosome depletion (Fig. 3B). The highly transcribed genes display the increased level of DNA-encoded nucleosome positioning signals at the close downstream of the TSS NDR (Fig. 3B). Regarding TTS, a dramatic discrepancy with experimental results is: top 25% genes with high transcription levels have a greatly increased nucleosome occupancy level at the near upstream of TTS than other genes (Fig. 3B). It is unclear why this discrepancy occurs.

The correlation of nucleosome positioning patterns at different types of promoters with gene expression was investigated in several studies [18, 26]. Lantermann et al. [26] used a clustering algorithm to identify eight promoter clusters with distinct nucleosome positioning patterns, which differed primarily in the depth and position of the NDR upstream of the TSS. Based on the distinct promoter clusters, the authors found that higher expression levels were correlated with deeper NDR troughs and lower promoter nucleosome occupancy [26]. Moyle-Heyrman et al. [47] also observed this trend by combining chemical nucleosome mapping with RNA-seq data. In line with these studies, our analysis of the chemical nucleosome map combined with Nanopore dRNA-seq gene expression data revealed the same pattern.

In addition, we are more interested in elucidating the principles or factors that determine nucleosome positioning in *S. pombe*. It was shown that when trained on *S. pombe* or *S. cerevisiae* data separately, a sequence-dependent model called N-score accurately predicted NDRs in the respective training species, but showed poor performance in cross-species prediction [26]. In contrast, our deformation energy model is not trained on species-specific nucleosome data, but is instead established purely on DNA physical properties. The model successfully recovers NDRs in *S. pombe*, even when applied to distinct gene types, thereby underscoring the importance of deformation-energy-related properties for nucleosome positioning in *S. pombe*. Furthermore, our results indicate that the transcription directionality mechanism cannot explain the upstream nucleosome arrays observed at tRNA TSSs.

## Sequence-independent nucleosome positioning determine the splice site usage

**Fig. 4:**
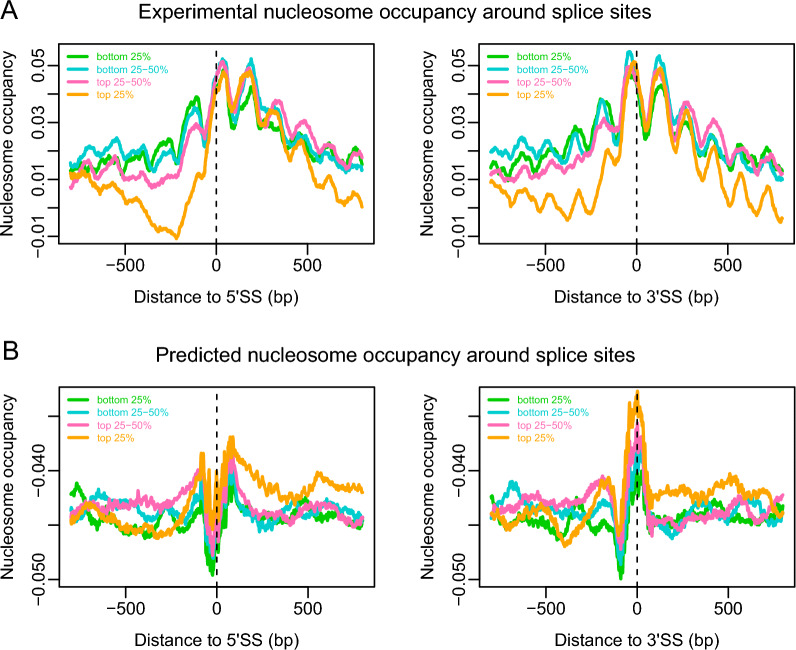
Nucleosome positioning pattern around RNA splice sites and its relationship with gene transcription level in *S. pombe*. (**A**) experimental occupancy based on the data from Moyle-Heyrman et al. [47], (**B**) predicted occupancy

The temporal and spatial coupling of transcription and RNA splicing, termed co-transcriptional splicing, enables the splicing process to be regulated by chromatin structure-level factors, including nucleosome positioning [16, 38]. Although important progress has been made toward understanding the regulatory mechanism of the co-transcriptional splicing, issues such as species-specific mechanisms and the roles of nucleosome in alternative splicing remain elusive. Here we explored the effect of nucleosome positioning on splicing and alternative splicing. A direct analysis of nucleosome occupancy around splice sites, stratified by gene transcription level, revealed that both 5' splice sites (5' end sites of introns, 5'SS) and 3' splice sites (3' end sites of introns, 3'SS) were preferentially occupied by nucleosomes. These nucleosome peaks are flanked by phased nucleosome arrays whose phasing and positioning signal decay with distance from the center (Fig. 4A). The center of the nucleosome peak at 5'SS is located mildly downstream the 5'SS, while the one at 3'SS is located mildly upstream the 3'SS. Moreover, the highly transcribed genes are characterized with strikingly decreased nucleosomes at the upstream regions of both 5'SS and 3'SS, accompanied by disappeared or reduced nucleosome phasing (Fig. 4A). It can be inferred that, similar to mammals, nucleosome enrichment at splice sites in *S. pombe* would be beneficial to efficient splicing, but the upstream devoid of nucleosomes is likely to assist RNA pol II elongation and up-reguate the transcription level. Interestingly, this orchestrated nucleosome pattern at splice sites is unlikely to be directed by DNA, as the results obtained by our DNA deformation energy-based model is incompatible or even contrary to experimental observation (Fig. 4B). Particularly, the nucleosome arrays around splice sites are not determined by sequence preference. In this regard, the role of chromatin remodeling factors in RNA splicing might be a promising direction worthy of further investigation.

We performed additional analyses considering different lengths of introns and exons (Figure S5–S7). Nucleosomes are enriched at splice sites regardless of intron length (Figure S5). Moreover, nucleosome phasing is much clearer relative to the 3′SS than to the 5′SS (Figure S5A), indicating that the 3′SS serves as a better reference point for positioned nucleosomes. When introns with long (> 150 bp) and short (< 150 bp) flanking exons are analyzed separately, results similar to those in Fig. 4A are observed: highly expressed genes show decreased nucleosome occupancy upstream of the 5′SS (Figure S6). Furthermore, nucleosome arrays are still present downstream of the 3′SS for both introns with both long and short flanking exons (Figure S6). When introns are stratified by intron length and flanking exon length, both nucleosome enrichment at splice sites and the surrounding nucleosome arrays remain evident (Figure S7). All these results indicate that the nucleosome positioning pattern around splice sites is independent of intron or exon length and not affected by whether introns or exons are too short to accommodate a nucleosome.

**Fig. 5:**
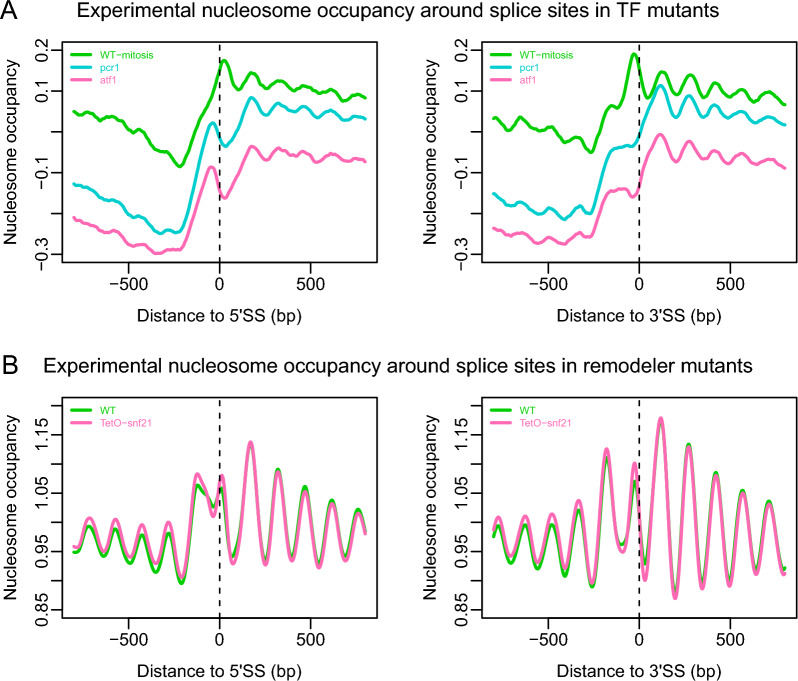
Alteration of nucleosome positioning around RNA splice sites in *S. pombe* gene mutants. (**A**) mutants of two transcription factors are compared with wild-type; (**B**) mutant of remodeler Snf21 is compared with wild-type. Experimental nucleosome occupancy was derived from GSE84912

By reanalyzing published data (GSE41773), we demonstrated the role of two transcription factors in remodeling the nucleosomes around splice sites. Firstly, in the experimental nucleosome maps for two TF mutants deficient in either *pcr1* or *atf1*, the nucleosome arrays around splice sites are disrupted. The closest nucleosome peak downstream the 5'SS (we call this 5'SS + 1 nucleosome) is totally abolished and an upstream nucleosome peak is established in the mutants (Fig. 5A). It appears that the 5'SS + 1 nucleosomes are displaced to 5'SS upstream. The location, phasing and spacing of nucleosomes from the 5'SS + 2 nucleosomes present in wild-type *S. pombe* are largely unaffected in the mutants. Secondly, the closest nucleosome upstream the 3'SS (we call this 3'SS + 1 nucleosome) is abolished in the mutants, and the 3'SS downstream nucleosomes moved mildly towards 3'SS (Fig. 5B). Thirdly, the overall nucleosome occupancy level around both 5'SS and 3'SS is reduced in the mutants. All these observations suggest that these two transcription factors (Pcr1 and Atf1) are essential determinants of nucleosome positioning patterns around splice sites, thereby regulating splice site usage and isoform production. In addition, a NDR region is established in the mutants where the 5'SS + 1 nucleosome or 3'SS + 1 nucleosome is positioned in the wild-type. The two transcription factors, Pcr1 and Atf1, belong to the ATF/CREB family of transcription factors and often function as a heterodimer. It was reported that by binding to CRE elements (consensus sequence: TGACGT) located within promoter regions of genes involved in environmental stress response, Pcr1 and Atf1 may sterically hinder nucleosome assembly, thereby promoting local chromatin accessibility [59]. Combining these implications with our results, we propose a model in which Pcr1 and Atf1 bind to genomic regions adjacent to splice sites, potentially serving as physical barriers that facilitate the formation of regular downstream nucleosome arrays. Importantly, within these arrays, the + 1 nucleosome positioned immediately downstream of the 5′ splice site may enhance splicing efficiency through a transcription-coupled mechanism. Supporting this hypothesis, our motif enrichment analysis identifies a consensus motif—ATGACGTA—enriched at the 5′SS (CentriMo, p = 1.5e-34), which contains the core binding sequence recognized by Pcr1 and Atf1 (Figure S8).

As indicated in our deformation energy-based prediction, the NDR region is intrinsically unfavorable for nucleosome positioning, suggesting again that trans-acting factors, such as nucleosome-displacing transcription factors and remodelers, rather than DNA sequence preference, is a major determinant of nucleosome positioning at splice sites, and DNA sequence preference becomes dominant once trans-acting factors are inactive. We also analyzed another nucleosome map, and found that the upstream regions of 5'SS and 3'SS may also form nucleosome arrays, despite a reduced signal as compared to downstream nucleosome arrays (Fig. 5B). The positions of the + 1 nucleosome at the 5' and 3' splice sites (Fig. 5B) are consistent with those observed in the other two maps (Fig. 4A, Fig. 5A). The *snf21* mutant shows no clear difference from the wild-type, suggesting that remodeler Snf21 is not involved in nucleosome positioning around splice sites (Fig. 5B).

Note that there must be some other factors regulating the nucleosome pattern around splice sites. In order to search for potential candidates, we first identified enriched motifs around splice sites using MEME [3]. A motif enrichment method called CentriMo [4] was used. Because there is no background TF-binding motif database of *S. pombe* available in the MEME yet, we used motif database of budding yeast (YEASTRACT 20130918) in the analysis, and then transcription factors known to target the enriched motifs were used as query to search for orthologs in *S. pombe*. Top five motifs enriched around 5'SS and 3'SS were presented respectively (Fig S9). Our results show that Mot3 and Reb1 preferentially bind motifs enriched at 5'SS, while Yap1, Yap3, Cad1, and Cin5 bind those enriched at 3'SS. Interestingly, the motif TTACTAA is enriched at the closely upstream intronic region of 3'SS (Fig S9). It should be noted that the TFs correspond to budding yeast, rather than fission yeast. Therefore, through database search (https://www.pombase.org/data/orthologs/), we found the orthologs of the TFs in fission yeast: Pap1 (ortholog of Yap1), Reb1 (ortholog of Reb1), and Eta2 (also an ortholog of Reb1). In other words, these TFs are likely to bind to the motifs in fission yeast and play a potential role in shaping nucleosome positioning pattern around 5'SS and 3'SS. This prediction needs further study to evaluate.

**Fig. 6:**
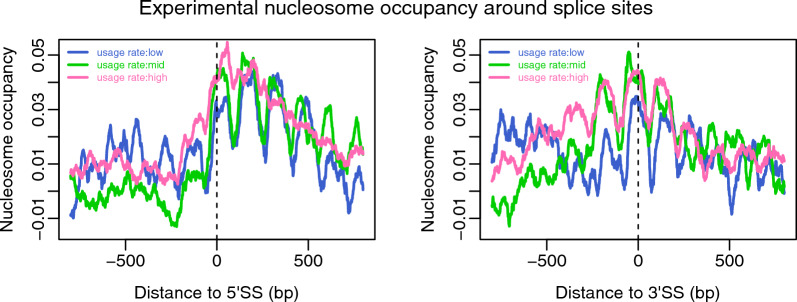
Experimental nucleosome positioning around splice sites and its association with splice site usage. Splice sites were categorized as low (≤ 0.5), medium (0.5–0.9), or high (≥ 0.9) according to usage rate. Nucleosome occupancy was computed using the data from Moyle-Heyrman et al. [47]

To further investigate the possible role of nucleosome positioning in alternative splicing, splice sites were separated into three groups according to their usage rate (see Methods for definition). Note that, in light of the proposed role of nucleosome in RNA splicing [30, 38], the basic rationale, underlying the link, if any, between nucleosome and alternative splicing is: nucleosome-assisted stay of splicing factors at splice sites improves splicing efficiency. Therefore, the splice site usage rate, defined as the proportion of transcripts in which a given splice site is used for splicing among all transcripts produced from a gene, is a possible index to infer nucleosome’s impact on alternative splicing. This is the reason why we analyzed the relationship between splice site usage rate and nucleosome positioning, instead of investigating the nucleosome organization for distinct alternative splicing modes. We observed that more frequently used splice sites (both 5'SS and 3'SS) were more preferentially occupied by nucleosomes (Fig. 6). Sequence-dependent predictions do not display clear nucleosome enrichment across splice sites grouped by their usage rates (Fig S10), suggesting again the sequence-independent property of nucleosome positioning at splice sites. All the results support that higher nucleosome occupancy at splice sites, which is shaped by non-DNA determinants, contributes to higher splicing efficiently, and variable splicing efficiency leads to alternative splicing.

**Fig. 7:**
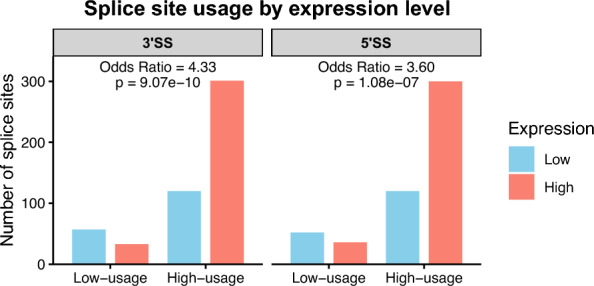
Distribution of splice site usage across expression levels. Bar plots illustrate the counts of 5' and 3' splice sites classified as low-usage (usage rate < 0.5) or high-usage (usage rate > 0.5) in low-expression and high-expression transcripts. Odds ratios and p-values from Fisher’s exact test are shown in each panel, demonstrating significant enrichment of high-usage sites in highly expressed transcripts for both 5'SS and 3'SS

As shown above, we see that splice sites of highly transcribed genes have substantially reduced nucleosome occupancy level at their upstream region (Fig. 4A), while the splice sites with high usage rate have elevated occupancy of nucleosomes around the splice sites (Fig. 6). Furthermore, high usage rate splice sites are enriched more in highly expressed genes, whereas the low usage rate splice sites are enriched more in lowly expressed genes (Fig. 7). This is consistent with the previous finding that splicing efficiency is high for highly expressed genes [68].

**Fig. 8:**
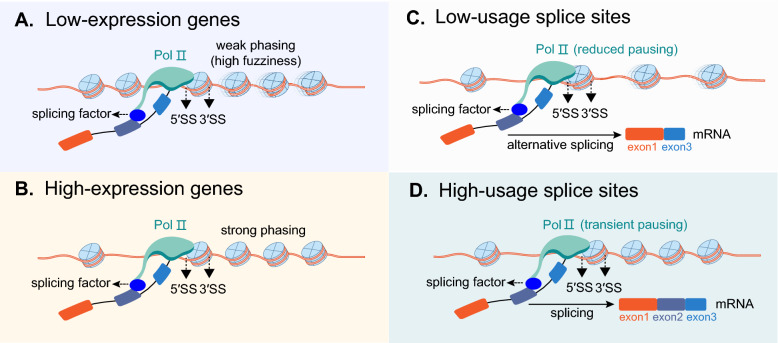
A hypothetical model for the biological relationships between nucleosome positioning, gene expression level, and RNA splicing. **A** Lowly expressed genes are characterized with high nucleosome occupancy upstream of splice sites and downstream weak nucleosome phasing; **B** highly expressed genes are characterized with low nucleosome occupancy upstream of splice sites and strong nucleosome phasing; **C** low-usage splice sites are characterized with low nucleosome occupancy around the spice sites; **D** high-usage splice sites are characterized with high nucleosome occupancy and downstream strong nucleosome phasing

As a summary for this part, a hypothetical model illustrating the biological relationships between nucleosome positioning, gene expression level, and RNA splicing is shown in Fig. 8. In this model, In contrast to lowly expressed genes, highly expressed genes exhibit lower nucleosome occupancy upstream of splice sites. Additionally, low-usage splice sites are associated with low nucleosome occupancy, whereas high-usage splice sites are associated with high nucleosome occupancy. Our model builds upon the RNA polymerase II elongation rate-mediated splicing mechanism [38] and the barrier effect of nucleosomes on transcriptional elongation [6]. We provide clear evidence for the strongest nucleosome peak located on the intronic side of splice sites and 3'SS-referenced downstream nucleosome phasing in *S. pombe*. This finding is in overall agreement with the preferential positioning of nucleosomes at exon–intron junctions in some organisms such as humans, flies and worms [58]. However, it markedly differs from these organisms in the location of the strongest nucleosome peak near splice sites: the strongest peak in *S. pombe* is located on the intronic side, whereas in previously studied species, the peak is found on the exonic side [2, 48, 58].

## Chromatin medium-range interaction might regulate transcription-coupled splicing

The roles of local chromatin environments, such as DNA methylation, and histone modifications, on splicing have been increasingly demonstrated, but it is largely unclear if splicing is regulated by chromatin interaction or 3D genome architecture. Previous studies revealed the roles of 3D genome architecture in development and diseases [28, 74, 76], but little is known about its potential role in the splicing process. A short article [1] discussed the hypothesis based on a few evidence supporting the relationship between interactions of exons with promoters and enhancers and alternative splicing events [41]. However, it is still unclear how 3D genome structure regulates splicing in diverse organisms, including *S. pombe*. In order to explore this issue, we first analyzed Hi-C captured intra-chromatin interactions around splice sites in *S. pombe*. Notably, since the Hi-C data were analyzed at a resolution of 1 kb, a bin size larger than all introns in *S. pombe*, each bin typically encompasses both the 5' and 3' splice sites (5'SS and 3'SS) of an intron. Therefore, we only present the results for the 5'SS, as they are representative of the 3'SS as well. The averaged interaction heatmap around splice sites indicated that the 5'SS were depleted of interactions (Fig S11A).

Depletion of Cut14, a subunit of condensin known to shape 3D genome structure in *S. pombe* [22], also resulted in a pattern very similar to that of the wild-type (Fig S11B), suggesting that Cut14 is not involved in the 3D genome structure around splice sites. We next asked whether the 3D genomic organization around splice sites differs between genes with different transcription levels. A comparison of averaged heatmaps revealed no apparent difference between low and high expression groups (Fig S11:C-D). Similarly, no obvious difference was observed in the averaged interaction heatmaps when comparing splice site groups with distinct usage rates (Fig S11:E–F).To investigate potential contacts between splice sites, we visualized the aggregated interactions for splice site pairs located 10–100 kb apart. Interestingly, enriched interactions were observed between the splice site-centered regions (Fig S12). No apparent difference in the interaction signal was observed between highly expressed and lowly expressed genes (Fig S12).

We focused on TSS-centered regions in the following analyses for two reasons. First, ~ 51% of protein-coding genes in *S. pombe* are intron-less. Focusing solely on splice site-centered regions would exclude these genes, whereas comparing intron-containing and intron-less genes could provide insights into the role of 3D genome in RNA splicing. Second, Hi-C data analysis generally requires target site-centered regions spanning > 20 bins (e.g. > 20 kb region around TSS in case of 1-kb bin size), and a range that typically encompasses all splice sites of a *S. pombe* pre-mRNA.

**Fig. 9:**
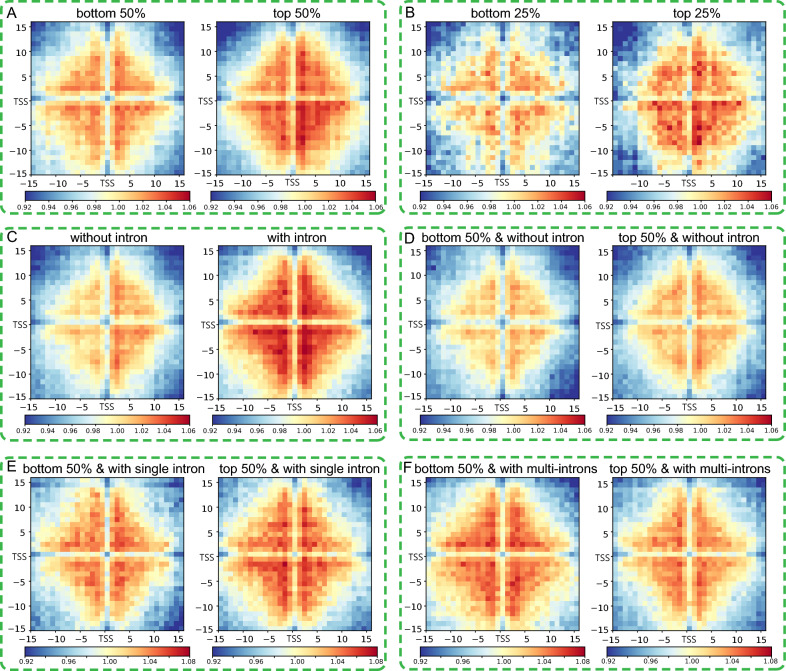
Aggregated contacts between TSS-centered regions. For the sake of reducing background strong short-range contacts, the range of contacts considered here was set to 10–100 kb. The bin size is 1 kb, and 31 bins centered at TSSs were used in the heatmaps. For each pair of analyzed TSS-centered regions, the Hi-C matrix derived from GSE143338 was converted into an observed/expected matrix. All submatrices corresponding to contacts between these regions were then aggregated to generate a single average matrix. **A** Bottom 50% vs. top 50%; **B** bottom 25% vs. top 25%; **C** comparison of genes with and without introns; **D** comparison of intron-less genes between bottom 50% and top 50% genes; **E** comparison of single-intron genes between bottom 50% and top 50% genes; **F** comparison of multi-intron genes between bottom 50% and top 50% genes

When we analyzed interactions between TSS-centered regions, we similarly observed enriched contacts surrounding TSSs, and this interaction was substantially stronger for highly expressed genes than for lowly expressed genes (Fig. 9A-B). Intriguingly, intron-containing genes exhibited much stronger contacts between TSS-centered regions than intronless genes (Fig. 9C). Among intron-less genes, highly expressed ones showed slightly increased inter-gene contacts compared to lowly expressed ones (Fig. 9D). At this point, one might expect that highly expressed intron-containing genes would exhibit stronger inter-gene interactions than their lowly expressed counterparts. However, our results showed that this expectation held true only for single-intron genes (Fig. 9E). In contrast, multi-intron genes displayed stronger interaction signals in lowly expressed genes than in highly expressed genes (Fig. 9F). Collectively, these results suggest that highly expressed genes without introns or with a single intron engage in more medium-range (10–100 kb) interactions than their lowly expressed counterparts. In contrast to highly expressed single-intron genes, highly expressed multi-intron genes are characterized by reduced inter-gene interactions, suggesting a distinct gene regulatory mechanism. Our explanatory model is as follows: highly expressed intron-poor genes, such as intronless and single-intron genes, engage in enhanced medium-range (10–100 kb) chromatin interactions, enabling their co-localization within sub-nuclear environments. These environments, like transcription factories, are enriched in transcriptional machinery such as RNA polymerase II, as well as splicing factors required for intron processing. By clustering together, these genes can share limited transcriptional and splicing resources, facilitating high expression levels. In contrast, multi-intron genes, due to their greater intron number, require more complex and time-consuming splicing that demands sustained availability of splicing factors. Given that splicing factors are relatively scarce within transcription factories, simultaneous high expression of multiple multi-intron genes would lead to resource competition. Consequently, highly expressed multi-intron genes do not rely heavily on clustering within factories; instead, they shift toward intragenic chromatin interactions, such as looping between the transcription start site and the terminator. This “inward” interaction mode coordinates transcription and splicing over long gene bodies, ensuring efficient expression while reducing dependence on intergenic factory localization. Thus, multi-intron genes optimize their own transcriptional-splicing coupling through internal looping, whereas intronless or single-intron genes leverage external interactions to share nuclear resources. The proposed model is based on the transcription factory model [62]. The transcription factory model proposes that transcription in eukaryotic nuclei occurs at discrete, specialized sites where multiple active RNA polymerase II (RNAPII) molecules are concentrated and anchored to a nuclear substructure. Active genes can cluster together by chromatin looping and share the same factory, potentially enabling co-regulation. Our model is not totally equivalent to the transcription factory model, but enriched the model by coupling transcription to splicing. Indeed, some studies suggested that genes with high transcription levels have preferred positions within *S. pombe* nucleus [19, 62], which supports our model. It is possible that the previously found small domains of ~ 50 kb [43, 63] might associate with the gene-proximal interactions observed in this study. Our model is also consistent with the finding that highly transcribed genes are characterized with high splicing efficiency [60, 69].

We also attempted to explore the association between chromatin interactions around splice sites and splice site usage. However, the resolution of the Hi-C data used in this study (~ 1 kb) was insufficient for this analysis. This limitation arises because a single gene often contains both high-usage and low-usage splice sites, the interactions specific to each group cannot be distinguished at 1 kb resolution.

## Roles of DNA physical properties in nucleosome positioning

**Fig. 10:**
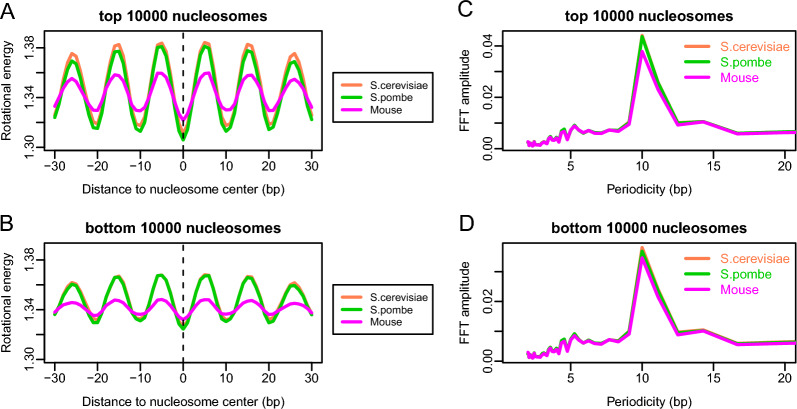
Comparison of bending energy-implicated rotational positioning signal of nucleosomes between different species. For the three species analyzed, both top 10,000 and bottom 10,000 nucleosomes with extreme NCP/noise signals were taken from experimentally determined unique nucleosome maps [8, 47, 67]. (**A**), bending energy profile for top 10,000 unique nucleosomes with high positioning scores. (**B**), bending energy profile for bottom 10,000 unique nucleosomes with low positioning scores. (**C**), FFT-based periodic signals in bending energies of top nucleosomes. (D), FFT-based periodic signals in bending energies of bottom nucleosomes. Bending energies were aligned at nucleosome centers and averaged across nucleosomes shown in (**A**) and (**B**). FFT was performed on the central 100 bending energy values for each nucleosome, and the resulting periodic signals were then averaged across the nucleosomes shown in (**C**) and (**D**)

How does DNA-encoded nucleosome positioning signal differ between species? According to our model, the 10-bp periodic oscillation of bending energy is a strong indicator of bending anisotropy, thereby suggesting the rotational positioning signal. We compared bending energy profile of unique nucleosomes between *S. cerevisiae*, *S. pombe*, and mouse ESCs. For each species, both top 10,000 and bottom 10,000 nucleosomes with extreme NCP/noise signals were taken from experimentally determined unique nucleosome maps [8, 47, 67]. Our results indicate that the top nucleosomes have increased magnitude of DNA bending potential than bottom nucleosomes (Fig. 10). In addition, compared to mouse genome, the genomes of both yeasts evolved to favor stronger rotationally-positioned nucleosomes (Fig. 10). No clear difference in the rotational positioning signal was detected between the two yeast species (Fig. 10). It is possible that if the nucleosome center positions are not identified precisely, the averaging of bending energies over the center-aligned nucleosomes would compromise the oscillation pattern of bending energies and affect the conclusions. To test this possibility, we use align-free Fast Fourier Transform (FFT) to analyze the 10-bp periodicity in bending energy, where high 10-bp periodicity indicates strong rotational positioning. Our results are consistent with the aforementioned conclusions (Fig. 10C-D). Furthermore, the FFT results from the concatenated energy sequence of multiple nucleosomes (Fig S13) are consistent with the averaged FFT results from individual energy sequences of nucleosomes (Fig. 10C-D). According to these results, we conclude that the DNA bendability required for nucleosome formation is nearly identical between *S. cerevisiae* and *S. pombe*, although *S. pombe* exhibits increased frequency of A/T-containing dinucleotides in nucleosomal regions compared to *S. cerevisiae* (Fig. S14).

**Fig. 11:**
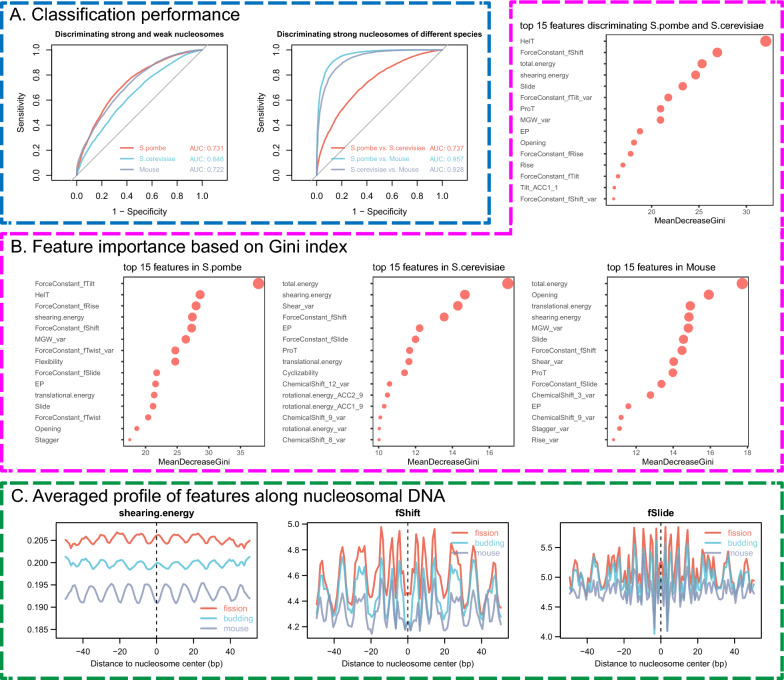
DNA property-based classification of within-species nucleosomes and between-species nucleosomes. For the three species analyzed, strong and weak nucleosomes refer to the top 10,000 and bottom 10,000 nucleosomes with the highest and lowest NCP/noise signals, derived from unique nucleosome maps [8, 47, 67]. (**A**), ROC-based classification performance. (**B**), feature importance evaluated by Gini index. The first three panels are most discriminative features between strong and weak nucleosomes in each species, and the last one lists the most discriminative features of strong nucleosomes between *S. pombe* and *S. cerevisiae*. (**C**), the average profiles of several DNA shear-related features along strong nucleosomes

In order to explore in-depth the possible species-specific roles of DNA physical properties in nucleosome positioning in fission yeast, we discriminated strong nucleosomes from weak nucleosomes using a Random Forest model, in which diverse DNA physical property features incorporated (see Methods). The strong and weak nucleosomes in *S. pombe* are best distinguished by DNA physical property features (Fig. 11A, left panel). Between-species predictions for strong nucleosomes showed that they could be successfully discriminated between *S. pombe* and mouse, but were least distinguishable between *S. pombe* and *S. cerevisiae* (Fig. 11A, right panel). Feature importance analyses based on Gini index showed that some features, such as shearing energy and electrostatic potential in the minor groove (EP) are consistently important for distinguishing nucleosome strength for the three species (Fig. 11B). However, the average level of DNA shear deformability differs substantially between the three species (Fig. 11C). For example, *S. pombe* shows the highest level of shearing energy, while *S. cerevisiae* and mouse have much lower level of shearing energy. Moreover, shearing energy is also one of the most discriminative features between *S. pombe* and *S. cerevisiae* (Fig. 11B). Furthermore, two shear deformability-related features, namely the force constants for shift and slide, in *S. pombe* are greater than the other two species (Fig. 11C).

The above machine learning analysis suggests that the average level of shearing energy is closely associated with nucleosome positioning strength (e.g. nucleosome occupancy) in *S. pombe*, as well as in *S. cerevisiae*. But, the shearing energy-based nucleosome occupancy model, which achieved a good performance in budding yeast [31, 34], was unsuccessful in predicting nucleosome pattern around TSS in fission yeast (Fig S4), as well as mouse ESCs [33, 35]. In addition, the results presented in this study suggest that DNA bending property, rather than DNA shear deformation, is an important indicator of locations of NDR near TSS and flanking nucleosome arrays in *S. pombe*. Collectively, our results give an interesting implication: DNA bending energy dominates the positioning mode of nucleosomes around gene ends (e.g. phasing), particularly the NDR near TSS and nucleosome arrays downstream the NDR in *S. pombe*, whereas DNA shearing energy is a better determinant of nucleosome strength. Nucleosome strength here represents nucleosome occupancy (e.g. the percentage of a genomic region being occupied by nucleosomes across a cell population). We therefore propose that, in *S. pombe*, the initial positioning of nucleosomes around the TSS is governed, at least in part, by anisotropic DNA bendability, whereas the percentage of genomic sites occupied by nucleosomes (nucleosome occupancy) across a cell population is modulated by DNA shear deformability.

## Conclusions

Our results indicate that DNA deformability is highly predictive of chemical-mapping determined nucleosome positioning patterns in *S. pombe*. Specifically, DNA bending energy governs, at least in part, the nucleosome depletion at transcription start sites and the downstream nucleosome arrays, whereas DNA shearing energy more accurately predicts nucleosome positioning strength. These findings extend the biophysical model of chromatin organization beyond sequence-based preferences, demonstrating that the intrinsic mechanical properties of DNA are important determinants of nucleosome architecture. This suggests that evolutionary selection on promoter and gene body regions may act not only on linear sequence motifs but also on DNA shape and flexibility.

We also establish connections among non-DNA factors, splice site usage, and chromatin organization. Nucleosome enrichment at splice sites is influenced by trans-acting factors, such as the transcription factors Pcr1 and Atf1, rather than intrinsic DNA sequence preferences, and correlates positively with splice site usage rate. Highly transcribed genes exhibit reduced nucleosome occupancy upstream of splice sites, whereas frequently used splice sites show enhanced nucleosome occupancy. Furthermore, we observed enhanced medium-range chromatin contacts (10–100 kb) between highly transcribed intron-poor genes compared to random expectation, potentially reflecting their preferential aggregation in sub-nuclear environments that facilitate efficient transcription and co-transcriptional splicing. Together, these results reveal a tripartite connection among transcription activity, splice site choice, and chromatin architecture.

Several limitations of this study point to future research directions. First, our DNA deformation energy model does not account for histone variants, histone modifications, or DNA methylation, all of which modulate nucleosome stability and positioning in vivo. Second, the interplay between DNA deformability and trans-acting factors such as transcription factors and chromatin remodelers, in regulating nucleosome dynamics remains to be elucidated. Third, experimental validation of the role of predicted transcription factors in splice site-associated nucleosome positioning is required. Finally, higher-resolution three-dimensional chromatin mapping will help to confirm how long-range chromatin interactions mechanistically contribute to co-transcriptional splicing.

## Supplementary Information

Supplementary Material 1

## Data Availability

Public data from GEO re-analyzed in this study include: GSE41773, GSE84912, and GSE143338. The code and data for the calculation of rotational deformation energy and nucleosome occupancy for the *S. pombe* genome are provided at github (https://github.com/gqliu1010/S.pombe_nucleosome). We also developed a webserver for the deformation energy model (http://bioinformatics.gqliulab.com/Nuc_DeformEnergy/) used in this study.
