# Supplementary material for: Contributions of DNA mechanics and trans-regulation to nucleosome positioning in Schizosaccharomyces pombe and its role in co-transcriptional splicing
Source: Epigenetics Chromatin. 2026 Apr 21;19:24. doi: 10.1186/s13072-026-00675-0 (PMC13255473; doi:10.1186/s13072-026-00675-0)
Supplement: Supplementary file 1 — Supplementary Material 1 [file 13072_2026_675_MOESM1_ESM.docx]

**Supplementary information for:**

**Contributions of DNA Mechanics and Trans-Regulation to Nucleosome Positioning in *Schizosaccharomyces pombe* and Its Role in Co-transcriptional Splicing**

Guoqing Liu^1,2,*^, Jing Cang^1^, Zhihao Du^1^, Xiangjun Cui^1,2^, Hongyu Zhao^1,2^, Jia Liu^3^

^1^ School of Life Science and Technology, Inner Mongolia University of Science and Technology, Baotou, China

^2^ Inner Mongolia Key Laboratory of Life Health and Bioinformatics, Inner Mongolia University of Science and Technology, Baotou, China

^3^ School of Science, Inner Mongolia University of Science and Technology, Baotou, China

*To whom correspondence should be addressed. Email: [gqliu1010@163.com](mailto:gqliu1010@163.com)

**Figure Captions**

**Figure S1.** Prediction of rotational positioning of top 10,000 nucleosomes in *S. pombe* based on DNA rotational energy. The prediction error denotes the distance between the experimentally-determined nucleosome center position and the position with the minimum deformation energy in the interval [−5, +5] around the experimental nucleosome center. The y-axis shows the percentage of prediction error.

**Figure S2.** Comparison of nucleosome positioning patterns between tRNA TSSs with and without bidirectional promoters in the 500 bp upstream region. Of the tRNA genes, 39 have bidirectional promoters and 132 do not.

**Figure S3.** Comparison of predicted and experimental nucleosome occupancy around TSS for several representative genes. Rotational energy-based model was used in the prediction. A cubic spline algorithm was used to smooth the predicted nucleosome occupancy. For direct comparison, min-max normalization was applied to both experimental and predicted nucleosome occupancy profiles, rescaling values to the [0, 1] range.

**Figure S4.** Comparison of predicted and experimental nucleosome occupancy around TSS. (A), around TSS of all transcripts; (B), around TSS of protein-coding genes; (C), around TSS of high confident transcripts defined in Lantermann et al. 2010. Four models were used in the prediction: translational energy-based model (Liu et al. 2021), shearing energy-based model (Liu et al. 2016), the model of Kaplan et al. 2009, and the NuPoP developed in Xi et al. 2010.

**Figure S5.** Experimental nucleosome positioning patterns around introns of different lengths. (A), around different-length introns; (B), around introns whose lengths were normalized to a uniform length of 100 bp.

**Figure S6.** Experimental nucleosome positioning patterns around introns from variably expressed genes with long (>150 bp) and short (<150 bp) and flanking exons at both ends. (A), introns with long (>150 bp) flanking exons; (B), introns with short (<150 bp) and flanking exons.

**Figure S7.** Experimental nucleosome positioning patterns around different-length introns with long (>150 bp) and short (<150 bp) and flanking exons at both ends. (A), introns with long (>150 bp) flanking exons; (B), introns with short (<150 bp) and flanking exons.

**Figure S8.** Enrichment of the Pcr1/Atf1-binding motif around 5'SS in *S. pombe*.

**Figure S9.** Top five motifs enriched around 5'SS and 3'SS in *S. pombe*.

**Figure S10.** Predicted nucleosome positioning pattern around RNA splice sites with distinct usage rate.

**Figure 11.** Chromatin interaction heatmaps around splice sites and its association with gene expression level and splice site usage rate. WT denotes wild-type, and MT denotes mutant. (A), wild-type cells; (B), Cut14-depleted cells; (C), low expression genes; (D), high expression genes; (E), low usage rate; (F), high usage rate.

**Figure S12.** Aggregated contacts between 5'SS-centered regions. For the sake of reducing background strong short-range contacts, the range of contacts considered here was set to 10-100 kb. The bin size is 1 kb, and 31 bins centered at 5'SSs were used in the heatmaps. For each pair of analyzed 5'SS-centered regions, the Hi-C matrix was converted into an observed/expected matrix. All submatrices corresponding to contacts between these regions were then aggregated to generate a single average matrix. (A), 5'SS from bottom 50% genes with low expression; (B), 5'SS from top 50% genes with high expression.

**Figure S13.** FFT amplitude for the concatenated energy sequence of multiple nucleosomes. (A), top 10,000 nucleosomal sequences; (B), bottom 10,000 nucleosomal sequences.

**Figure S14.** Comparison of the frequency of A/T-containing dinucleotides (AA/TT/AT/TA) in nucleosomal regions between *S. pombe* and *S. cerevisiae.*

**Table S1.** Chemical shift parameters of atoms on the DNA sugar-phosphate backbone

**Figure S1.** Prediction of rotational positioning of top 10,000 nucleosomes in *S. pombe* based on DNA rotational energy. The prediction error denotes the distance between the experimentally-determined nucleosome center position and the position with the minimum deformation energy in the interval [−5, +5] around the experimental nucleosome center. The y-axis shows the percentage of prediction error.

**Figure S2.** Comparison of nucleosome positioning patterns between tRNA TSSs with and without bidirectional promoters in the 500 bp upstream region. Of the tRNA genes, 39 have bidirectional promoters and 132 do not.

**Figure S3.** Comparison of predicted and experimental nucleosome occupancy around TSS for several representative genes. Rotational energy-based model was used in the prediction. A cubic spline algorithm was used to smooth the predicted nucleosome occupancy. For direct comparison, min-max normalization was applied to both experimental and predicted nucleosome occupancy profiles, rescaling values to the [0, 1] range.

**Figure S4.** Comparison of predicted and experimental nucleosome occupancy around TSS. (A), around TSS of all transcripts; (B), around TSS of protein-coding genes; (C), around TSS of high confident transcripts defined in Lantermann et al. 2010. Four models were used in the prediction: translational energy-based model (Liu et al. 2021), shearing energy-based model (Liu et al. 2016), the model of Kaplan et al. 2009, and the NuPoP developed in Xi et al. 2010.

**Figure S5.** Experimental nucleosome positioning patterns around introns of different lengths. (A), around different-length introns; (B), around introns whose lengths were normalized to a uniform length of 100 bp.

**Figure S6.** Experimental nucleosome positioning patterns around introns from variably expressed genes with long (>150 bp) and short (<150 bp) and flanking exons at both ends. (A), introns with long (>150 bp) flanking exons; (B), introns with short (<150 bp) and flanking exons.

**Figure S7.** Experimental nucleosome positioning patterns around different-length introns with long (>150 bp) and short (<150 bp) and flanking exons at both ends. (A), introns with long (>150 bp) flanking exons; (B), introns with short (<150 bp) and flanking exons.

**Figure S8.** Enrichment of the Pcr1/Atf1-binding motif around 5'SS in *S. pombe*.

**Figure S9.** Top five motifs enriched around 5'SS and 3'SS in *S. pombe*.

**Figure S10.** Predicted nucleosome positioning pattern around RNA splice sites with distinct usage rate.

**Figure 11.** Chromatin interaction heatmaps around splice sites and its association with gene expression level and splice site usage rate. WT denotes wild-type, and MT denotes mutant. (A), wild-type cells; (B), Cut14-depleted cells; (C), low expression genes; (D), high expression genes; (E), low usage rate; (F), high usage rate.


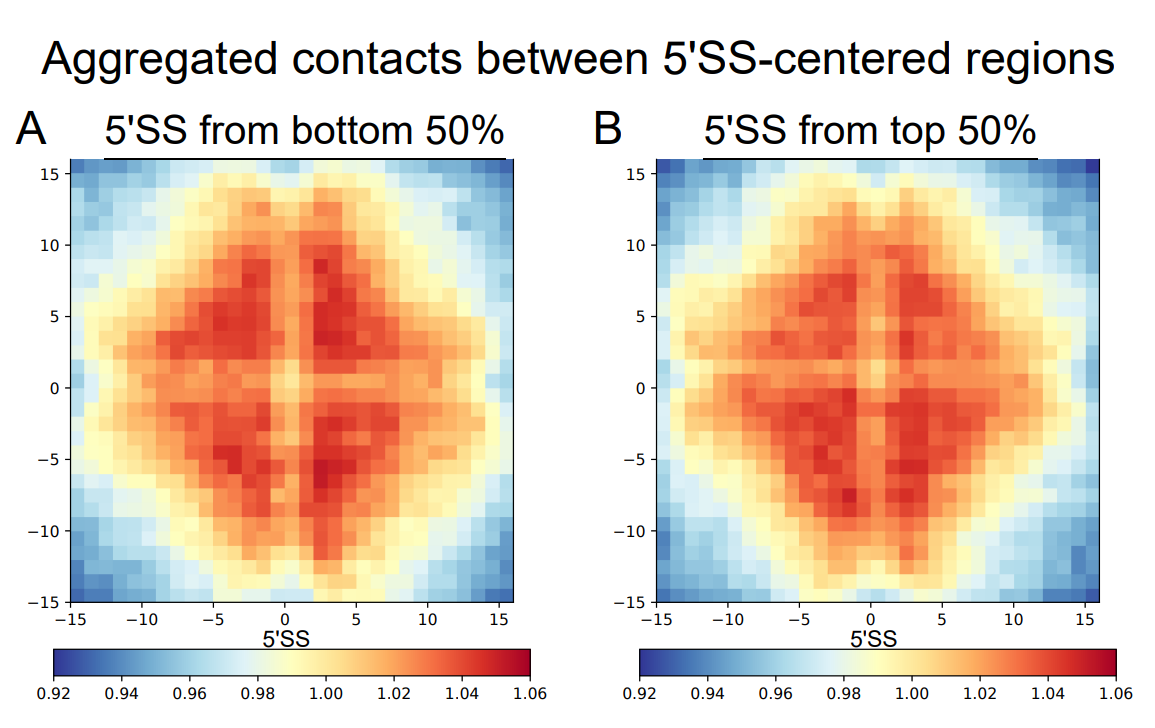


**Figure S12.** Aggregated contacts between 5'SS-centered regions. For the sake of reducing background strong short-range contacts, the range of contacts considered here was set to 10-100 kb. The bin size is 1 kb, and 31 bins centered at 5'SSs were used in the heatmaps. For each pair of analyzed 5'SS-centered regions, the Hi-C matrix was converted into an observed/expected matrix. All submatrices corresponding to contacts between these regions were then aggregated to generate a single average matrix. (A), 5'SS from bottom 50% genes with low expression; (B), 5'SS from top 50% genes with high expression.


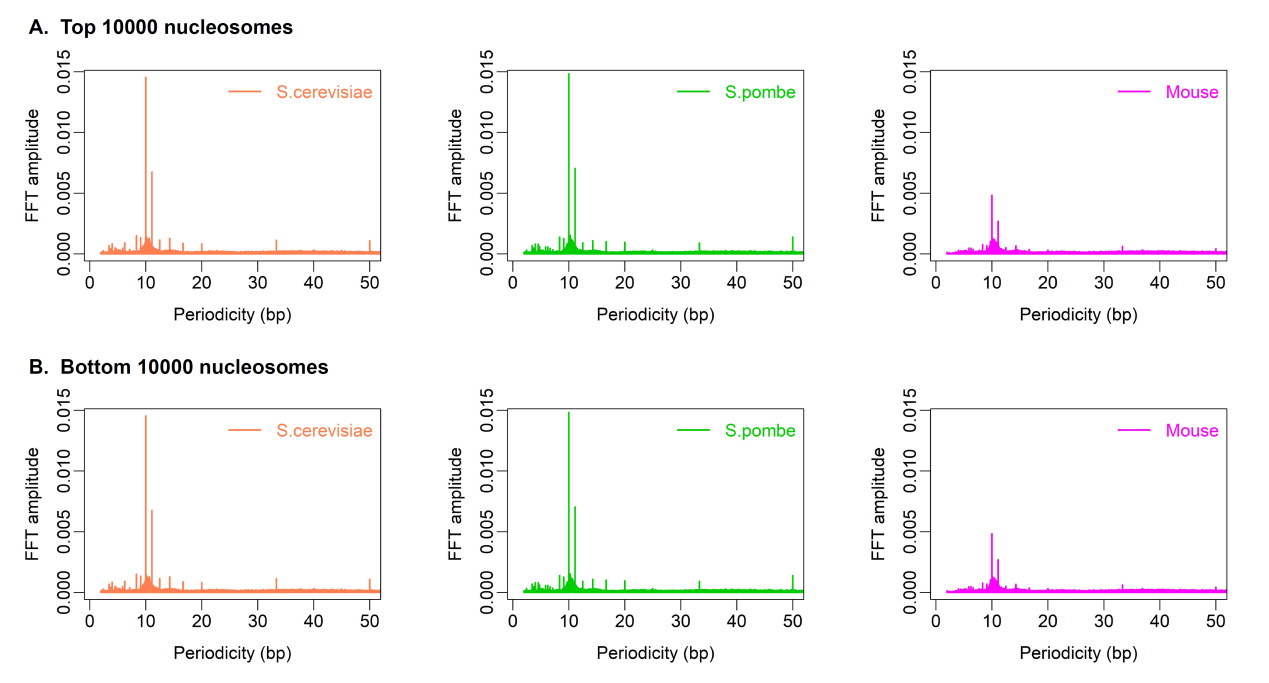


**Figure S13.** FFT amplitude for the concatenated energy sequence of multiple nucleosomes. (A), top 10,000 nucleosomal sequences; (B), bottom 10,000 nucleosomal sequences.

**Figure S14.** Comparison of the frequency of A/T-containing dinucleotides (AA/TT/AT/TA) in nucleosomal regions between *S. pombe* and *S. cerevisiae.*

**Table S1.** Chemical shift parameters of atoms on the DNA sugar-phosphate backbone

| atom_id | H1' | H2' | H2'' | H3' | H4' | H5' | H5'' | C1' | C2' | C3' | C4' | C5' | P |
| --- | --- | --- | --- | --- | --- | --- | --- | --- | --- | --- | --- | --- | --- |
| A | 6.079 | 2.62 | 2.772 | 4.96 | 4.351 | 4.057 | 4.034 | 85.114 | 40.287 | 78.443 | 86.732 | 66.583 | -1.717 |
| G | 5.919 | 2.611 | 2.678 | 4.934 | 4.353 | 4.125 | 4.1 | 84.91 | 39.945 | 78.148 | 86.684 | 66.851 | -1.79 |
| C | 5.855 | 2.056 | 2.363 | 4.755 | 4.138 | 4.015 | 3.999 | 86.987 | 40.173 | 77.11 | 85.725 | 65.926 | -1.662 |
| T | 5.941 | 2.099 | 2.36 | 4.785 | 4.142 | 3.963 | 3.959 | 86.899 | 39.892 | 77.339 | 85.796 | 66.495 | -1.966 |
